# Exercise and Arterial Modulation in Children: The EXAMIN YOUTH Study

**DOI:** 10.3389/fphys.2019.00043

**Published:** 2019-02-01

**Authors:** Katharina Endes, Sabrina Köchli, Lukas Zahner, Henner Hanssen

**Affiliations:** Department of Sport, Exercise and Health, Medical Faculty, University of Basel, Basel, Switzerland

**Keywords:** childhood cardiovascular risk, primary prevention, physical activity, retinal vessel diameters, arterial stiffness

## Abstract

**Background:** Cardiovascular disease (CVD) remains to be one of the most frequent causes of death worldwide. Cardiovascular (CV) risk factors such as hypertension and obesity often manifest in childhood. The study examines the associations of blood pressure, body mass index and physical activity with cardiopulmonary, metabolic, and psychosocial health of children in a systems physiology approach.

**Methods/Design:** This cross-sectional study will be performed in a cohort of 6 to 8 year old school children (*n* = 1000). As a measure of vascular health, retinal microvascular diameters and large artery pulse wave velocity will be examined. Anthropometric parameters, such as weight, height, body mass index, and blood pressure will be assessed according to standardized protocols for children. Physical fitness and activity will be measured by a 20 m shuttle run, a 20 m sprint and a proxy-reported questionnaire on lifestyle behavior. Spirometry, assessment of heart rate variability and skin advanced glycation end products as well as a flanker test will be performed to determine systemic end organ alterations.

**Discussion:** The study offers a unique integrative primary prevention concept that aims to set the grounds for a healthy and active lifestyle approach during childhood. It will help optimize CV risk stratification to identify children at risk of disease progression later in life. The study will demonstrate the importance of specific CV screening programs in children to reduce the growing burden of CV disease in adulthood. Prospective follow-up studies will have to prove the efficacy of primary prevention programs in children to achieve healthier aging as a long-term goal.

## Background

Cardiovascular disease (CVD) is a chronic smoldering inflammatory disease of the vascular bed including coronary heart disease and cerebrovascular disease. Myocardial infarction and stroke are the first and second leading causes of death worldwide and are responsible for more than 30% of global deaths World Health Organization, 2014. Two of the most crucial risk factors for cardiovascular (CV) morbidity and mortality are high blood pressure (BP) and obesity (Kontis et al., 2014). Hypertension and obesity are becoming a health risk with an increasing prevalence of unhealthy and sedentary lifestyle already among children (Broyles et al., 2015; Seidell and Halberstadt, 2015). The yearly progression rate of prehypertension to hypertension is stated as being about 7% (National Heart Lung and Blood Institute, 2012). The longitudinal CV risk in Young Finns Study found that elevated BP as a child tracks into adulthood (Oikonen et al., 2016). A systematic review of the United States Preventive Services Task Force highlighted the necessity to screen for hypertension in children and adolescents in order to prevent CVD in adulthood (Thompson et al., 2013). Epidemiological surveys of children and adolescents in the past 20 years have found an increase in the prevalence of prehypertension and hypertension, which could be attributed to a concomitant increase of overweight and obese children (Falkner, 2010; National Heart Lung and Blood Institute, 2012). Almost every fourth child in the Western world is overweight or obese (Ng et al., 2014). All obese children are at risk of becoming obese as an adult (Freedman et al., 2001) and to suffer from CVD as an adult (Baker et al., 2007; Bibbins-Domingo et al., 2007; Twig et al., 2016). Bibbins-Domingo et al. (2007) based on adolescent’s overweight in the year 2000, the prevalence of obesity in men is expected to increase from 30 to 37% and in women from 34 to 44% between the years 2000 and 2020. The increased obesity rate leads to an estimated increase of the incidence of coronary heart disease by a range of 5 to 16% in the year 2035. Physical inactivity (PIA) is one of the main risk factors not only for the development of obesity and hypertension but also for the high prevalence of CVD. The World Health Organization has stated that more than 75% of CV mortality can be prevented by lifestyle changes (World Health Organization, 2009; Diaz and Shimbo, 2013). Physical activity (PA) and cardiorespiratory fitness (CRF) are important for the prevention and treatment of CVD in adults, as well as for the prevention of obesity, high BP, and CV risk factors in children and adolescents (Hillsdon et al., 2005; Janssen and Leblanc, 2010; World Health Organization, 2014). Increases in PA and physical fitness can significantly reduce CV mortality, independent of BP, and body mass index (BMI) (Paffenbarger et al., 1986; Blair et al., 1996; Kujala et al., 1998). A 20-year follow up study demonstrated a 35% lower mortality risk for unfit individuals who improved their fitness level compared to the ones who stayed unfit (Kokkinos et al., 2010). High BP is directly linked to modifiable lifestyle factors such as PIA. Moderate aerobic exercise of 60–90 min per week, for example, can reduce systolic BP by about 12 mmHg and diastolic BP by 6–8 mmHg in adults (Ishikawa-Takata et al., 2003). It therefore seems necessary to implement primary prevention programs to optimize risk stratification in childhood to prevent development of CVD later in life.

### Childhood Cardiovascular Health: A Systems Physiology Approach

It was found that childhood obesity is related to early signs of atherosclerosis (Berenson, 2002). It appears to be important to find vascular biomarkers that are closely linked to obesity-related risk factors and hypertension in children. Retinal vessels share the embryological origin and morphological as well as physiological properties with the cerebral vasculature and are considered a biomarker of cerebrovascular disease in adults (Patton et al., 2005). A very recent meta-analysis has demonstrated the use of retinal vessel abnormalities as a surrogate marker for ischemic cerebrocascular disease (Dumitrascu et al., 2018). Retinal vessels are regulators of local retinal blood flow, and as such, they are valid and robust microvascular surrogate biomarkers of CV risk. Retinal vascular signs have therefore been postulated to be a window to the heart (Flammer et al., 2013). In addition, retinal vessel analysis allows the investigation of alterations of the venous structure and function (phlebosclerosis) in the microcirculation.

Changes in the microcirculation seem to appear before common CV risk factors. A recent meta-analysis has shown that childhood obesity and hypertension are associated with retinal microvascular abnormalities. A higher BMI is associated with retinal arteriolar narrowing and wider retinal venular diameters while systolic and diastolic BP are associated with narrower retinal arteriolar diameters (Kochli et al., 2018). PA and PIA have also been shown to affect retinal vessel diameters. Hanssen et al. (2012) found that PIA was related to a lower arteriolar-to-venular diameter ratio (AVR) in children due to a wider central retinal venular equivalent (CRVE). Gopinath et al. (2011) have shown an association between PA and wider central retinal arteriolar equivalent (CRAE). Furthermore, we have previously found (Imhof et al., 2016) an association between higher CRF and narrower CRVE and a higher AVR.

In adults, large cohort studies have found that smaller CRAE, larger CRVE and a resulting lower AVR correlate with an increased risk and seriousness of hypertension (Wang et al., 2003; Wong et al., 2004a; Ikram et al., 2006b), a higher risk of stroke (Ikram et al., 2006a; McGeechan et al., 2009b) and a higher CV morbidity and mortality in the older population (Wong et al., 2002; Wang et al., 2007). Given the predictive value of changes in microcirculation for CVD cases in adults (Wang et al., 2006; McGeechan et al., 2009a,b), early identification of subclinical alterations in the retinal vessel diameters in children seems to be indispensable to improve primary prevention of CVD. The role of the microcirculation for the progress of CVD is less clear compared to findings of the macrocirculation. CVD progression is directly linked to the long-term asymptomatic process of arteriosclerosis (Cohn, 2006). Arteriolosclerosis is a condition that comprises arterial hardening and thickening. Arterial stiffness consists of two main components: a structural arterial sclerosis and a functional stiffness based on the contractility of the vessels’ smooth muscle cells. In a subclinical phase of arteriosclerosis, the aorta and major arteries stiffen, and constantly lose their elastic and distensible abilities (Wang and Fitch, 2004).

Arterial stiffness is most frequently measured by pulse wave velocity (PWV) and it is commonly acknowledged as the “gold-standard” method for examining central/aortic arterial stiffness (Laurent et al., 2006). Central PWV is an independent predictor for CV morbidity and mortality, stroke and all-cause mortality in the general population, elderly subjects and in patients with hypertension, end-stage renal disease, and diabetes mellitus (Blacher et al., 1999; Laurent et al., 2001; Meaume et al., 2001; Sutton-Tyrrell et al., 2005; Mattace-Raso et al., 2006; Willum-Hansen et al., 2006). In a systematic meta-analysis of 17 longitudinal studies evaluating more than 15 000 individuals over 7.7 years, Vlachopoulos et al. (2010) found that an increased arterial stiffness is associated with a twofold increase in CV events and mortality for persons with high compared to low aortic PWV. An increase of PWV by 1 m/s stands for a risk increase of fifteen percent in CV and all-cause mortality (Vlachopoulos et al., 2010).

It has been reported that obesity and hypertension are related to higher central arterial stiffness in adults (Brunner et al., 2015; Wen et al., 2015). The beneficial effects of PA and physical fitness on arterial stiffness have been shown in adults with obesity and hypertension (Madden et al., 2009; Montero et al., 2014a,b). Studies that examined arterial stiffness in children are scarce, but childhood obesity and elevated BP seem to be associated with increased PWV (Sakuragi et al., 2009; Urbina et al., 2010; Yilmazer et al., 2010). Low childhood PA has been associated with higher arterial stiffness in children (Veijalainen et al., 2016). Improving CRF by increasing PA levels in adults seems to contribute to a lower incidence of CVD and mortality, which may also be related to a decrease of arterial stiffness (Ferreira et al., 2003).

Microvascular and macrovascular complications have been linked with an increased concentration of subcutaneous advanced glycation end products (AGEs) (Goldin et al., 2006; Ueno et al., 2008; Araszkiewicz et al., 2011). AGEs form after the interaction of proteins or lipids with sugars for a prolonged period of time. They undergo molecular transformations to form glycated proteins or lipids, which induces distinct, and maladaptive remodeling in the vessel wall (Schmidt et al., 1994; Brownlee, 1995; Singh et al., 2001). A recent review in children found that AGEs are involved in the pathogenesis of adiposity and β-cell failure (Gupta and Uribarri, 2016).

Cardiovascular mortality is associated with malfunctioning of the autonomic nervous system (Shaffer et al., 2014). The most promising marker of autonomic activity is the HRV (Task Force of the European Society of Cardiology the North American Society of Pacing Electrophysiology, 1996; Shaffer et al., 2014). Decreased HRV has been associated with cardiac arrhythmia, obesity, hypertension, Type 1 and 2 diabetes and psychological disorders in adults (Thayer and Sternberg, 2006; Henry et al., 2010; Schmid et al., 2010; Boer-Martins et al., 2011) and in children (Akinci et al., 1993; Baharav et al., 1999; Xie et al., 2013; Santos-Magalhaes et al., 2015).

**Figure 1:**
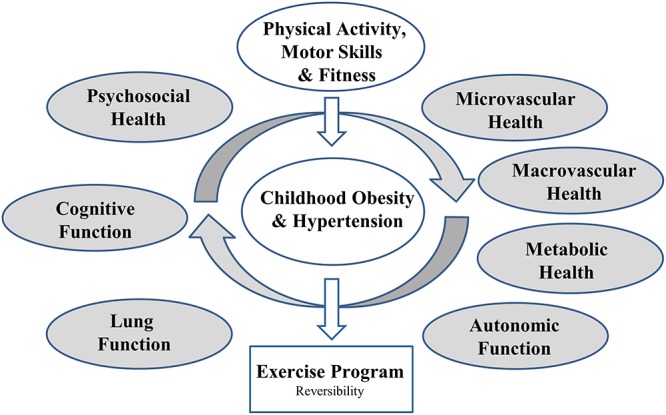
EXAMIN YOUTH study concept.

In addition to CV biomarkers, obesity has also shown to be associated with reduced lung function, caused by fat deposits pressing on the chest and abdominal cavities in adults (Beuther and Sutherland, 2007; Salome et al., 2010; Torun et al., 2014; Frey et al., 2015). Together with the growing number of overweight and obesity in children, pulmonary diseases are becoming increasingly frequent (Beuther et al., 2006; Rzehak et al., 2013; Frey et al., 2015). While individuals with obesity tend to be less active and prone to respiratory disease, people with respiratory disease often avoid PA leading to obesity. Overall, obesity and respiratory alterations are mutually reinforcing entities (Beuther and Sutherland, 2007; Egan et al., 2013; Gratas-Delamarche et al., 2014). Classical CV risk factors such as BP, BMI and PIA are interrelated with target organ damage of multiple systems such as the cardiopulmonary, metabolic and central nervous system. Therefore, the role of BP, BMI, and PIA for the development of chronic disease during childhood needs to be studied in a systems physiology approach (Figure 1).

### Aims

Aim 1: To assess the interrelation of BP, BMI, and CRF/PA with retinal vessel diameters and arterial stiffness.

Aim 2: To assess the association of BP, BMI, and CRF/PA with AGEs and HRV.

Aim 3: To assess the interrelation of BP, BMI, and CRF/PA with pulmonary and cognitive function.

Aim 4: To assess the interrelation of BP, BMI, and CRF/PA with psychosocial health.

### Outcome Measures

Primary outcome: CRAE.

Secondary outcomes: CRVE and AVR.

Further outcomes: CRF and PA, PWV, peripheral and central BP; central pulse pressure (cPP); AGEs; HRV; lung function; flanker test (reaction time and accuracy).

### Hypotheses

Hypothesis 1: BP and BMI are associated with retinal arteriolar narrowing, retinal venular widening and higher arteriolar stiffness.

Hypothesis 2: A higher CRF and PA level are associated with wider CRAE, narrower CRVE and lower arterial stiffness.

Hypothesis 3: High BP and BMI and low CRF/PA are associated with higher levels of AGEs, lower HRV, impaired pulmonary and cognitive function as well as adverse psychosocial health.

### Inclusion Criteria

All children aged 6-to-8-years of primary state schools in Basel-Stadt with an informed consent signed by their parents will be included.

### Exclusion Criteria

Primary school children with motor- or other disabilities that do not allow participation will be excluded.

## Materials and Equipment

### Retinal Vessel Analysis

Static retinal vessel diameters will be analyzed using a Static Retinal Vessel Analyzer (SVA-T, Imedos Systems UG, Jena, Germany). The system consists of a fundus camera (Topcon TRC NW8) and analyzing software (Visualis 2.80, Imedos Systems UG), allowing non-invasive assessment of retinal vessel diameters without mydriasis. Two valid images from the retina of the left and right eye with an angle of 45° and with the optic disk in the center will be taken on the day of the medical screening. Coursing through an area of 0.5–1 disk diameter from the optic disk margin, retinal arterioles. and venules will be identified semi-automatically at higher magnification using special analyzing software (Vesselmap 2, Visualis, Imedos Systems UG). Diameters are averaged to CRAE and CRVE, using the Parr-Hubbard formula described elsewhere (Hubbard et al., 1999). The AVR will be calculated as the ratio of CRAE over CRVE. Retinal vessel diameters are generally presented in μm. In the model of Gullstrand’s normal eye, 1 measuring units relates to 1 μm. Reliability of this method has been shown to be high, with inter-observer and intra-observer interclass correlation coefficients for arteriolar and venular diameter measurements ranging from 0.78 to 0.99 (Hubbard et al., 1999; Wong et al., 2004b). In our previous study with school children the correlation coefficient for CRAE, CRVE. and AVR ranged between 0.94 and 0.95 (*p* < 0.001 each) (Imhof et al., 2016).

### Pulse Wave Velocity (PWV)

Pulse wave velocity is a non-invasive and established measurement of arterial stiffness. Arterial stiffness is defined by the geometric and elastic properties of large arteries (Wassertheurer et al., 2010; Middeke, 2017). PWV will be examined using the validated oscillometric Mobil-O-Graph^®^ Monitor (I.E.M. GmbH, Germany) integrating ARCSolver software (Franssen and Imholz, 2010; Wassertheurer et al., 2010; Weiss et al., 2012). A slow PWV indicates low arterial stiffness. PWV will be calculated from the data of the pulse wave reflection and resembles an indirect measurement of central/aortic arterial stiffness. The oscillometric device estimates central systolic and diastolic BP and the cPP based on the measurement of the peripheral BP values. cPP resembles the systolic pressure maximum minus the diastolic pressure.

Pulse wave analysis will be performed in a supine position and appropriate small-sized cuffs are placed on the left upper arm. The first of two measurements will be taken after 5 min of rest to allow for calibration based on the systolic BP. Thereafter, two PWV measurements will be performed at 2-min intervals. After the measurements, data will be reviewed for quality and erroneous values according to the manufacture’s manual and as described previously (Wassertheurer et al., 2010). In case of poor data quality, further measurements will be performed to calculate the mean and standard deviation of at least two valid measurements.

### Advanced Glycation End Products (AGEs)

The AGE Reader (DiagnOptics Technologies BV, Groningen, Netherlands) will be used to detect and analyze the accumulation of AGEs in the connective tissue. AGEs will be measured by skin autofluorescence (SAF) with an integrated spectrometer. There is a strong correlation between AGEs (measured by SAF) in the connective tissue and those in the blood serum (measured by skin biopsies) (Mulder et al., 2006). SAF will be expressed in arbitrary units (au) and calculated as the ratio between the emission light (range between 420 and 600 nm) and reflected excitation light (range between 300 and 420 nm) multiplied by 100. Each child will be measured three times (triple measurement) at different areas on the right ventral side of the forearm and scores will be taken as the arithmetic mean.

### Heart Rate Variability (HRV)

Heart rate variability will be recorded with a 3-channel ECG (Custo flash, Custo med GmbH, Germany). In combination with the Custo diagnostic software, the ECG data will be recorded and evaluated. The ECG will be applied for 10 min in a supine position. Time- and frequency-based parameters will be used for further analysis. In order to be able to estimate the total HRV, the standard deviation of all NN intervals in the measuring range (SDNN) will be used. The root mean square of the successive NN interval differences (RMSSD), the percentage of successive RR intervals more than 50 ms (pNN50), and the high frequency power from the power density spectrum in the frequency range of 0.15–0.40 Hz (HF) will be used to estimate the parasympathetic activity. The low frequencies from the power density spectrum in the frequency range of 0.04–0.15 Hz (LF) predominantly reflect the sympathetic activity (Weiner and McGrath, 2017).

### Spirometry

Spirometry is a well-established, non-invasive method to measure lung volumes and lung capacities (Ferguson et al., 2000). Measurements of lung function will be assessed using a small handheld validated spirometer (EasyOne Diagnostic, Andover, MA, United States). The standardized examinations will be performed by experienced investigators. Objective parameters of respiratory function such as FEV1 (forced expiratory volume in 1 s), FVC (forced vital capacity), the ratio of FEV1/FVC (ratio of forced expiratory volume in 1 s to forced vital capacity), PEF (peak expiratory flow) and FEF25-75 (forced expiratory flow at 25 to 75%) will be measured. The FEV1/FVC ratio is considered to be a most specific parameter to identify airway obstructions, while FEV1 grades the severity of ventilatory defects (Jat, 2013). During the test, children will be placed in a supine position with their feet firmly on the floor. A nose clip will be worn whilst breathing through the disposable spirette (breathing tube). Before the start of the examination, the children will be asked to practice with the EasyOne spirometer until they will be able to perform the lung function test correctly. After a few calm breaths the child will have to exhale completely, inhale as much as possible, followed by a fast, prolonged expiration. The test will be repeated at least five times, whereby the most valid test will be used for statistical analysis. The results will only be rated as valid if the degree of quality was between grade A (minimum three acceptable attempts and difference of the two best FEV1- and FVC-values ≤100 ml) and C (minimum two acceptable attempts and difference of the two best FEV1- and FVC ≤ 200 ml) as defined in the EasyOne Manual (Ayer et al., 2011).

### Cognitive Function

Inhibitory control will be assessed with a computer-based version of the Flanker task, which is administered with E-Prime 2.0 (PST, United States) (Eriksen and Eriksen, 1974). Visual stimuli (subtending 1.8° visual angle in height) will be five black fish presented on white background. In congruent trials, the fish will be facing the same direction, whereas in incongruent trials the central fish will be facing in the opposite direction of the flanking fish. During the task, participants are required to press a button corresponding to the direction of the central fish (left/ right arrow on a keyboard). Following an inter-trial interval of 1100–1500 ms (random variation), the visual stimuli will be presented for 2000 ms or until a response will be collected. After participants complete 16 practice trials, two test blocks with 32 trials each will be administered. The blocks will be interspersed by a 30 s break. As dependent variables, reaction time (on response-correct trials) and accuracy are calculated separately for congruent and incongruent trials.

### Anthropometry

Standing height (without shoes) will be measured with a wall-mounted statiometer (Seca, Basel, Switzerland). Body weight and percentage body fat in light clothing will be measured using InBody 170, a bioelectrical impedance analyzer (InBody 170 Biospace device; InBody Co., Seoul, Korea). Based on recommendations of the American Heart Association, peripheral BP will be assessed after a rest period of 5 min at the bare right arm (Pickering et al., 2005). Children will be seated in a comfortable position in a quiet room. To reduce inter-observer variability, an automated oszillograph (Oscillomate, CAS Medical Systems, Branford, CT, United States) is used. Depending on the individual mid-upper arm circumference of the children, specific cuff-sizes for children will be fitted. BP measurements will be taken five times. For further analysis the mean of the three measurements with the smallest variation will be used (National High Blood Pressure Education Program Working Group, 2004). Central BP and the cPP will be measured by use of the oscillometric Mobil-O-Graph^®^ pulse wave reflection Monitor device as described above.

### Physical Fitness

Physical fitness will be assessed by a combination of the “Körperkoordinationstest für Kinder” (Kiphard and Schilling, 1970), the Eurofit test (Committee for the Development of Sport, 1988) and the “Allgemeiner Sportmotorischer Test für Kinder (AST 6-11) (Bös and Tittelbach, 2002). Included in the test battery will be CRF, strength and coordination. All tests will be performed during two normal school lessons on the site of the school with the same equipment. Before testing a standardized 5 min warm-up will be conducted. The following four tests will be included:

1. 20 m shuttle run test (Leger et al., 1988): This is a validated test (Van Mechelen et al., 1986) to measure CRF in children. Fitness will be measured by running back and forth over a 20 m distance. The initial running speed will be 8.0 km/h witha progressive raise of running speed by 0.5 km every minute. The pace is set by audio-beeps from a stereo. Numbers of stages (1 stage ≅ 1 min) performed until exhaustion will be counted with a precision of 0.5 stages.
2. 20 m sprint (Bös et al., 2001): Speed v measured using electronic time gates (precision: 1/100 s; HL2-31, Tag Heuer. La Chaux-de-Fonds, Switzerland) by running a 20 m sprint, starting from a resting position. The start follows after an acoustic signal released by the examiner. The faster of two trials will be used for further analysis.
3. Jumping sideward (Kiphard and Schilling, 1970): With this test speed and coordination will be measured. The task will be to jump with both legs together on alternating sides of a wooden beam, as many times as possible, within 15 s. Two trials will be performed. The number of jumps will be counted. The sum of the number of jumps of both tests will be used for statistical analysis.
4. Balancing backward (Kiphard and Schilling, 1970): By balancing backward on 3 m long bars, coordination will be examined. The bars will be 6 cm, 4.5 cm and 3 cm wide. Starting on the 6 cm bar, and ending on the 3 cm bar, the number of steps until the child’s foot is touching the ground will be counted. On every bar three trials will be performed. The sum of all nine trials will be used for statistical analysis.

The fitness and motor skill tests will be supervised by an experienced sports scientist. The instructions of all tests will be standardized. The order of the fitness tests will not be the same for every child, but the 20 m shuttle run test will be performed at the end of each test day, because this is the only exhausting test which may affect the other outcomes. The test of coordination and speed will be conducted in a randomized order as no influence on other test results are expected (Rosser et al., 2008).

### Questionnaires

The parents provide information about their socio-economic background and estimate the PA level of their children by questionnaire [using items from the Ballabeina study (Niederer et al., 2009)]. They will be asked for information about their children’s stress exposure and psychosomatic complaints. To assess stress exposure, a modified version of the 22-item Life Event Checklist (Johnson and McCutcheon, 1986) will be administered. The parents will report whether a specific event has occurred during the past 3 months and whether this event has negatively affected the well-being of the child from 1 (no effect at all) to 4 (strong adverse effect). As family issues are among the most important sources of distress among young children (Grant et al., 2003), family conflict will be assessed with the validated 9-item Conflict subscale of the Family Environment Scale (Moos, 1996). To assess their children’s mental health, parents will fill in the 8-item HBSC Symptom Checklist (Haugland and Wold, 2001). Additionally, parents will fill in the validated KINDL-R to assess children’s health-related quality of life (Ravens-Sieberer and Bullinger, 2004). Two validated questionnaires will be added to assess attention deficit hyperactivity disorder (Lidzba et al., 2013; Christiansen et al., 2016) and insomnia [lnsomnia Severity Index (Bastien et al., 2001)]. To eliminate language problems, the parental questionnaires will be translated from German in the other seven most spoken languages in Switzerland, i.e., French, Italian, Spanish, Portuguese, Serbian, Albanian, and Turkish (Statistisches Amt Basel-Stadt, 2010).

## Stepwise Procedures

### Study Design and Participants

**Figure 2:**
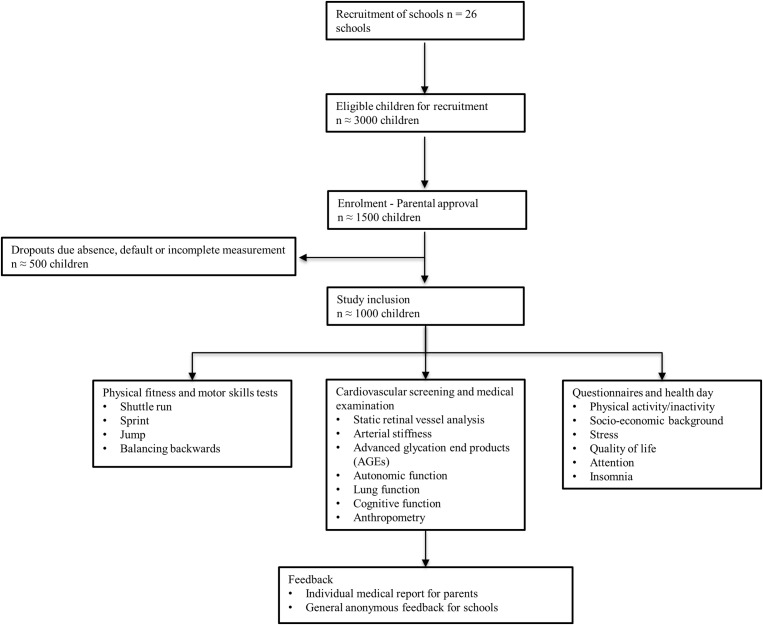
Flow diagram.

The study will be conducted in a cohort of 6 to 8 year old children to assess the association of BP, body composition and PA and fitness with vascular, pulmonary, metabolic, autonomic and cognitive dysfunction as well as psychosocial health (Figure 1). The study is designed as a large-scale cross-sectional study. All primary school children of Basel-Stadt will take part in the measurements of motor skills and physical fitness, body composition and height during physical education lessons (*n* = ∼1000). Measurements will be conducted at every school in Basel-Stadt (*n* = 26). Children with an informed written consent by their parents will be allowed to participate in the medical screening on a separate visit. Approximately 1000 children with complete data will be included in the study (Figure 2).

### Setting

This cross-sectional study will be realized by the Department of Sport, Exercise and Health, Basel, Switzerland and will be conducted at all primary school in Basel-Stadt, Switzerland. The study has been approved by the Ethics Committee of Northwestern and Central Switzerland (EKNZ, No.: 258/12) and has been registered on ClinicalTrials.gov (NCT02853747).

### Study Procedures and Ethical Considerations

All children and their parents receive information authorized by the Ethics Committee of Northwestern and Central Switzerland (EKNZ) containing details on methods of the study. Only children with prior written consent from their parents are to participate. Children and parents will be briefed about their right to reject from the study without implications. On the day of the medical screening, children will be again asked to give their oral agreement. In between the medical screening, which will be scheduled to take 1 h per child, children will be distracted by the option of drawing with pencils and paper in a separated supervised play area. Children with a medical certificate that prohibits PA will be excluded from physical fitness assessment. All measurements conducted in this study will be non-invasive and performed according to standardized procedures for children. Retinal vessels will be analyzed by use of a non-mydriatic fundus camera. The use of eye-drops will not be required. The study will be carried out in accordance with the principles stated in the Declaration of Helsinki and the Guidelines of Good Clinical Practice (GCP) (World Medical, 2013). This study protocol was design according the SPIRIT Guidelines.

### Proposed Analysis

#### Statistical Analysis

Participants’ characteristics will be presented using descriptive statistics. Variance homogeneity will be assessed using Tukey-Anscombe Plots, and to assess normality, normal QQ plots of the residuals are to be used. To analyze our primary outcome, the associations of BP, BMI, and CRF with retinal arteriolar diameter (CRAE) in 6–8 year old school children, a multiple linear regression analysis using CRAE as outcome will be conducted. The regression models will be adjusted for age and sex as well as BMI and BP. To compare clinical categories of BP (normotension, prehypertension and hypertension) and BMI (normal weight, overweight, obesity), univariate analysis of covariance (ANCOVA) will be used. Pearson’s correlation will be used to assess the correlation of micro- and macrovascular parameters. Statistical analyses will be performed using the computer software program Stata 15 (StataCorp LP, Texas, TX, United States) and α < 0.05 will be considered as statistically significant. Arithmetic means and confidence interval (CI) will be calculated for each variable.

### Sample Size Calculation

For the power calculations of the primary endpoint, we refer to the corresponding data in our previous Swiss cohort of children, where the linear multiple regression analysis showed a medium effect (*f*^2^ = 0.12) for the model analyzing the associations between BP and CRAE (Imhof et al., 2016). While we aim to achieve complete capture of all data from all participating children, it will be unavoidable that some participants will fail to provide outcome data or simply will not attend school on the day of the measurements. Applying the same statistical calculations as described in the previous publication (Imhof et al., 2016) but on a larger sample size of 1000 children at the same level of significance, we have a power of up to a 100% to detect an association between BP and CRAE with an increase in R2 of 0.10 or above.

### Anticipated Results

The anticipated results can be derived from the main hypotheses. We further anticipate pitfalls with respect to performing the study in the school setting as well as limitations of the methods applied.

### Potential Pitfalls

The medical screening will be conducted in school settings and not under laboratory conditions. The quality of the test procedures will be affected by the facilities available at the schools. To minimize confounding factors, test requirements and instructions during the test-period will be standardized assuring a high level of data quality. The medical screening and the fitness and motor skill assessments will be performed on separate days to allow for more comfortable timing and to ensure the measurements do not affect each other. On the day of the medical test, children are asked to remain fastened. They will receive a healthy small breakfast after the examinations. Each standardized measurement will be performed after at least 5 min of rest. In case of time restraints some children may not receive all of the measurements to remain within the scheduled screening time of 1 h per child and to ensure a high degree of data quality.

### Methodological Limitations and Considerations

The shuttle run test is not the gold standard test for the measurement of CRF. But Mayorga-Vega et al. (2015) found in their meta-analyses examining the validity of the 20 m shuttle run test that if a laboratory-based test is not feasible, the 20 m shuttle run test is a useful alternative for estimating CRF. In their meta-analysis, Mayorga-Vega et al. (2015) found that a 20 m shuttle run test is a useful and valid alternative for estimating CRF if a laboratory-based test is not feasible.

It is unclear if the link between micro- and microvasculature changes from childhood to adulthood. There is, however, a convincing amount of data demonstrating very similar associations of CV risk factors with retinal vessel diameters in children as compared to adults. This holds true for higher BMI as well as BP, with few data on PIA. A recent meta-analysis gives an overview on the available data and compared the findings in children with those in adults. It can be concluded that childhood retinal vessel diameters are a valid microvascular biomarker for CV risk in children and adults alike (Kochli et al., 2018).

Further, HRV is arguably a marker for cardiac autonomic function with several publications showing this link in adults by use of 24 h ECGs and PA and fitness is known to modulate HRV (Lahiri et al., 2008; Soares-Miranda et al., 2014). While these landmark publications have shown the relevance of HRV as a marker of cardiac autonomic function, other work has highlighted the inconsistency of the available data, specifically the use of frequency-domains (Taylor and Studinger, 2006; Picard et al., 2009; Monfredi et al., 2014). A fair amount of data is also available on HRV and autonomic function in children. One review looked at the influence of age and weight status on cardiac autonomic control in healthy children (Eyre et al., 2014). It is concluded that obesity may affect the normal maturation of cardiac autonomic function. A systematic review and meta-analysis has assessed the effects of exercise on HRV in healthy children (da Silva et al., 2014) but, according to their findings, exercise did not affect HRV in healthy children. It will be of utmost importance to look at the associations of physical fitness with HRV in subgroups such as children with obesity.

## Discussion and Conclusion

Childhood hypertension and obesity are becoming growing health hazards threatening to worsen the deleterious prevalence of adult CVD and the associated morbidity and mortality. The EXAMIN YOUTH cohort study offers a holistic primary prevention strategy for CV risk screening in early childhood. The study will investigate the influence of BP, BMI, and PA behavior on determinants of CV risk and subclinical impairments of physiological systems and end organ function. The research approach has a focus on the association of childhood BP and BMI with micro- and macrovascular health as biomarkers for subclinical atherosclerosis and the risk of developing CV disease later in life. We aim to clarify if higher PA and CRF are related to improved vascular health in young children. Since the detrimental effects of BP, BMI, and PIA in children are not restricted to vascular remodeling and vascular dysfunction, a systems physiology approach is warranted to optimize risk stratification in children. The assessment of pulmonary, metabolic, and psychosocial health as well as autonomic and cognitive function is included in the study design. Our study is characterized by an extensive phenotyping of childhood health in a large cohort allowing analysis of the interrelation of multiple CV risk factors (Figure 1). The proposed study aims to set an example for an integrative CV risk screening with the potential to deliver clinically relevant missing data to improve primary CV prevention in childhood. It links systems physiology with CV prevention, exercise medicine and clinical pediatrics.

It is a matter of ongoing debate, which non-invasive tissue biomarker best resembles underlying CV risk in age and disease. The study allows for the examination of pathophysiological links between the micro- and macrocirculation. So far, little is known about the cross-talk between the two vascular beds and how they influence one another. There is a need to examine early signs of vascular alterations and endothelial dysfunction in large as well as small arteries of the vascular bed. The retinal microcirculation is a new and innovative tissue biomarker for the assessment of CV risk and microvascular pathology in all age groups. On the basis of the current literature and our own preliminary work, it has been shown to be a valid and sensitive diagnostic tool to analyze underlying microvascular impairments in young children. Data on the association of physical fitness on micro- and macrovascular health and its interrelation with childhood health is scarce. Our study will increase our understanding of the complex mechanisms involved in the etiology and development of CVD.

Some general limitations of the present study remain to be discussed. Selection bias may occur due to the voluntariness of the study. The cross-sectional study design does not allow for analysis of causal relationship or temporal association. It remains to be elucidated whether impairments of the vascular bed or other forms of systems pathophysiology are predictive of the development of CVD later in life. A prospective follow-up study enables to assess the predictive value of vascular biomarkers for development of hypertension and CV risk during childhood development. It can help place the clinical relevance of our systems physiology assessment and risk stratification into perspective. The knowledge gained will contribute to a better understanding of CV disease development during childhood. In conclusion, the study aims to help define new recommendations for timely CV risk stratification in children and to bridge the gap from a screening approach to potential non-pharmaceutical and exercise treatment approaches of childhood hypertension and obesity. Classical and non-classical CV risk factors are associated with subclinical target organ damage such as retinal arteriolar narrowing and venular widening as well as increased arterial stiffness. Higher PA and fitness may have the potential to counteract the development of cardiopulmonary, metabolic and cognitive dysfunction in childhood, which may otherwise lead to manifest disease in adulthood. The study will demonstrate the potential of extensive CV screening programs and exercise interventions for young children to reduce the growing health hazard of obesity and hypertension and the associated increased long-term risk of manifest CVD. Healthy and active children with normal weight and normal BP are more likely to become healthy adults, which will eventually reduce the prevalence and burden of CVD in adulthood. As a future step, prospective follow-up studies will have to prove the predictive value of biomarkers for development of manifest CVD and the clinical efficacy of primary prevention programs in children to achieve healthier aging as a long-term goal.

## Author Contributions

KE wrote the manuscript, helped to design the study, and was the study coordinator of the fitness and motor skill tests. SK helped to write the manuscript, was the study coordinator of the medical examinations, and supervised the field workers. LZ helped to design the study and arranged the cooperation with the Department of Education of the City of Basel and revised the manuscript. HH designed the study, supervised KE and SK, and helped to write the manuscript. All authors read and approved the final manuscript.

## Conflict of Interest Statement

The authors declare that the research was conducted in the absence of any commercial or financial relationships that could be construed as a potential conflict of interest.

## Abbreviations

AGEs: advanced glycation end products
AVR: arteriolar-to-venular diameter ratio
BMI: body mass index
BP: blood pressure
cPP: central pulse pressure
CRAE: central retinal arteriolar equivalent
CRF: cardiorespiratory fitness
CRVE: central retinal venular equivalent
CV: cardiovascular
CVD: cardiovascular disease
FEF 25–75: forced expiratory flow at 25 to 75%
FEV1: forced expiratory volume in 1 s
FEV1/FVC: ratio of forced expiratory volume in 1 s to forced vital capacity
FVC: forced vital capacity
HRV: heart rate variability
PA: physical activity
PEF: peak expiratory flow
PIA: physical inactivity
PWV: pulse wave velocity
SAF: skin autofluorescence.
